# Common cardiac medications potently inhibit ACE2 binding to the SARS-CoV-2 Spike, and block virus penetration and infectivity in human lung cells

**DOI:** 10.1038/s41598-021-01690-9

**Published:** 2021-11-12

**Authors:** Hung Caohuy, Ofer Eidelman, Tinghua Chen, Shufeng Liu, Qingfeng Yang, Alakesh Bera, Nathan I. Walton, Tony T. Wang, Harvey B. Pollard

**Affiliations:** 1 Department of Anatomy, Physiology and Genetics, Uniformed Services University School of Medicine, Uniformed Services University of the Health Sciences, Bethesda, MD 20814 USA; 2 Collaborative Health Initiative Research Program (CHIRP), Uniformed Services University of the Health Sciences, Bethesda, MD 20814 USA; 3 Consortium for Health and Military Performance (CHAMP), Uniformed Services University of the Health Sciences, Bethesda, MD 20814 USA; 4 Laboratory of Vector-Borne Viral Diseases, Division of Viral Products, Center for Biologics Evaluation (CBER), U.S. Food and Drug Administration, Silver Spring, MD 20993 USA; 5 Center for the Study of Traumatic Stress (CSTS), Uniformed Services University of the Health Sciences, Bethesda, MD 20814 USA

**Keywords:** Target validation, Cystic fibrosis, Mechanism of action

## Abstract

To initiate SARS-CoV-2 infection, the Receptor Binding Domain (RBD) on the viral spike protein must first bind to the host receptor ACE2 protein on pulmonary and other ACE2-expressing cells. We hypothesized that cardiac glycoside drugs might block the binding reaction between ACE2 and the Spike (S) protein, and thus block viral penetration into target cells. To test this hypothesis we developed a biochemical assay for ACE2:Spike binding, and tested cardiac glycosides as inhibitors of binding. Here we report that ouabain, digitoxin, and digoxin, as well as sugar-free derivatives digitoxigenin and digoxigenin, are high-affinity competitive inhibitors of ACE2 binding to the Original [D614] S1 and the α/β/γ [D614G] S1 proteins. These drugs also inhibit ACE2 binding to the Original RBD, as well as to RBD proteins containing the β [E484K], Mink [Y453F] and α/β/γ [N501Y] mutations. As hypothesized, we also found that ouabain, digitoxin and digoxin blocked penetration by SARS-CoV-2 Spike-pseudotyped virus into human lung cells, and infectivity by native SARS-CoV-2. These data indicate that cardiac glycosides may block viral penetration into the target cell by first inhibiting ACE2:RBD binding. Clinical concentrations of ouabain and digitoxin are relatively safe for short term use for subjects with normal hearts. It has therefore not escaped our attention that these common cardiac medications could be deployed worldwide as inexpensive repurposed drugs for anti-COVID-19 therapy.

## Introduction

COVID-19 is a pandemic pneumonia-like disease, caused by the SARS-CoV-2 virus, that was first detected late in 2019 in Wuhan, China^1–3^. The virus initially gains access to type II pneumocytes in the lung when the Receptor Binding Domain (RBD) on the viral Spike (S) protein binds to the extracellular carboxypeptidase domain of the ACE2 receptor protein on the cell surface^4–6^. The native spike structure is composed of three identical protomers. Each protomer is divided into two domains: the distal N-terminal S1 spike "tip", containing the RBD, and the residual C-terminal S2, transmembrane fusion protein. In the intact Spike, the binding process appears to depend on the RBD in at least one protomer having transitioned from a down "closed" state to an upper "open" state^7,8^. In the down "closed" state the RBD has been shown by Cryo-EM to be partially hidden by glycosylated spike amino acids^9^. It has been proposed that the three RBDs in a spike may exist as an equilibrium of thermodynamically defined energy states where 0, 1, 2 or all 3 RBDs are elevated at any one time, and thus available to bind to ACE2^10^. However, the process by which contact between the RBD and ACE2 is first established remains to be fully understood.

Following binding of ACE2 to the Spike RBD, the Spike protein is cleaved by Furin, a host protease, into the distal S1 segment and the residual S2 segment^11,12^. Furin cleavage reveals the C-terminal *CendR* domain, to which the host protein neuropilin 1 binds and promotes cell entry^11^. The residual S2 domain is further sequentially optimized for membrane fusion by the host proteases TMPRSS2 and endosomal CTSL^6,9^. However, since the Original viral strain from Wuhan was first sequenced, the virus has mutated into more infectious and possibly more lethal strains. Using the WHO nomenclature, examples of such mutations in the spike include the mutation at residue 614, α/β/γ [D614G]^13,14^. This mutation appears to increase spike density and flexibility^15,16^. The mutation also creates an additional cleavage site for serine elastase 2 (neutrophil elastase) that promotes further exposure and priming of S2^6^. Additional mutations have been detected in the RBD, including the α/β/γ [N501Y]^17^; [Y453F], the Mink mutation^18^; and the β [E484K]^19^. The β [E484K] mutation contributes to recent COVID-19 outbreaks in India in the B.1.116 lineage, and in Brazil as variants P.1 and P.2 within the B.1.1.33 lineage. Since infection depends on successful viral entry into the cell, this complex entry mechanism has therefore become a promising target for anti-viral SARS-CoV-2 drug discovery.

In an effort to jumpstart COVID-19 drug discovery, unbiased large scale in silico and in vitro screens of FDA-approved and other drugs have been performed to find possible high affinity blockers of SARS-CoV-2 infectivity^20–22^. In some of these screens the steroidal cardiac glycoside drugs digitoxin, digoxin and ouabain, have emerged as top contenders. Experimentally, time-of- addition experiments with infection by native SARS-CoV-2 of green monkey kidney Vero cells have been interpreted to suggest that ouabain blocks virus penetration, while digoxin blocks infectivity at an unknown intracellular site^23^. Experiments with digoxin^24^ and digitoxin^25^ have also been interpreted as blocking entry of native MERS-CoV into target cells. Consistently, in silico docking sites for digitoxin have been identified in the RBD^26^. An in silico docking site for both digitoxin and digoxin has also been identified on the ACE2 receptor protein^27^. However, other in silico studies have shown that cardiac glycoside drugs might also interact with the active site of the viral main protease, Mpro^28^ or with a site on ACE2^27^. In silico simulations have also identified steroids as potential ligands on the Spike non-RBD free-fatty acid pocket^29^, and on two pocket domains on the RBD^30^. However, large scale screens of FDA-approved drugs and related compounds for docking on these latter three regions did not identify cardiac glycosides among potential ligands. Thus it remains to be determined where in the infectivity process cardiac glycoside drugs act, since in most in vivo studies with coronaviruses the experimental endpoints for cardiac glycoside action have been viral progeny counts.

Based on the foregoing, we hypothesized that cardiac glycoside drugs such as digitoxin, digoxin ouabain, might block the binding reaction between ACE2 and the viral Spike (S) protein, and thus block viral penetration into target cells (Supplemental Fig. S1). To test this hypothesis we first developed a biochemical method to measure the kinetics of ACE2 binding to immobilized variants of the Spike S1 and RBD domains. We found that binding occurred by a positively cooperative mechanism. Next we tested the cardiac glycoside drugs for their ability to block the ACE2:RBD binding reaction. These drugs were all found to be competitive inhibitors. The most potent was ouabain, followed by digitoxin, with digoxin being the least active. This relationship of inhibitory potencies was also observed when we tested, in vivo, whether these cardiac glycoside drugs could block viral entry into human lung cells with SARS-CoV-2-Spike-pseudotyped virus, and whether they could also block infectivity by native SARS-CoV-2. These cardiac glycoside drugs are inexpensive, widely available, and clinically safe for subjects with normal hearts^31–33^. It is therefore possible that these drugs could be repurposed for COVID-19 prevention and therapy.

## Results

### ACE2 binds with positive cooperativity to receptor binding domain proteins

**Figure 1:**
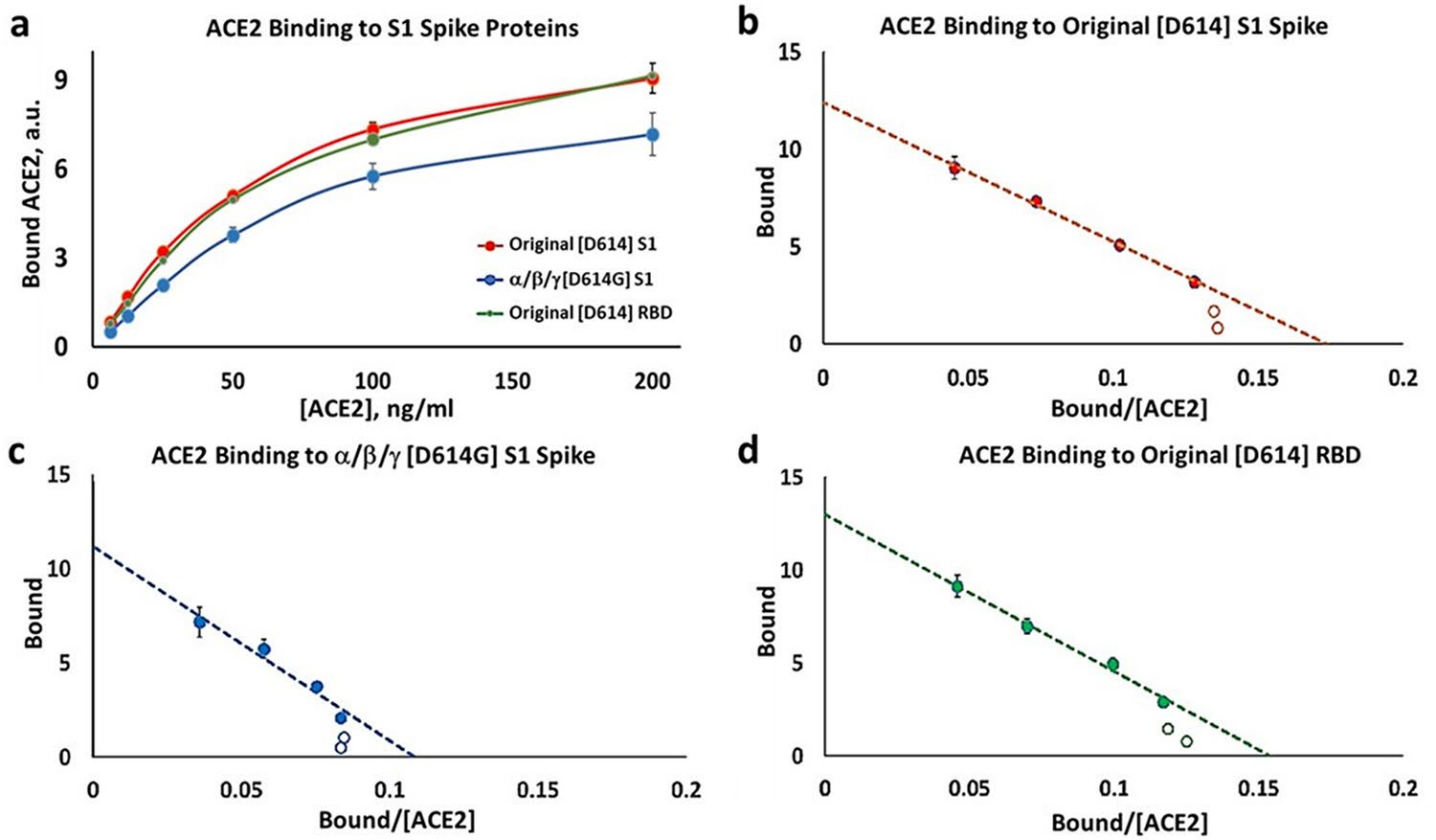
Binding of ACE2 to the SARS-CoV-2 Spike variants. (**a**) Substrate-Binding plots for original [D614] S1 spike (red), α/β/γ [D614G] S1 spike (blue), and original [D614] RBD (green) proteins. (**b**–**d**) Eadie–Hoffstee plots of data in *Part (a)* for original [D614] S1 spike (**b**), α/β/γ [D614G] S1 spike (**c**), and original [D614] RBD from the S1 spike (**d**). Note concave-down structure at low binding levels. Each point is the average ± SE for N = 5–6 independent experiments.

The substrate-binding plots in Fig. 1a show that both the Original [D614] RBD protein, and the larger Original [D614] S1 domain protein, appear to have very similar binding isotherms at 37 °C. However, the binding isotherm for the mutant α/β/γ [D614G] S1 differs quantitatively from that of the Original S1 and Original RBD. Furthermore, inspection of the substrate-binding plots show that a lag in binding occurs at low concentrations of ACE2. Thus, the binding isotherms appear to show a distinct sigmoid appearance in the low concentration range that is independent of mutation. To further test this possibility, we constructed Eadie–Hofstee (EH) plots for each of the domains. In the case of the Original [D614] S1 protein Fig. 1b shows a "concave-down" structure at the lower values of ACE2 binding. Such behavior is characteristic of positive cooperativity, including positive cooperativity by monomeric proteins or enzymes^34^. Figure 1c, d show the same descriptive positive cooperativity behavior in Eadie–Hofstee plots for both the α/β/γ [D614G] S1 protein and the Original [D614] RBD protein, respectively. Hill plots of these data also show the modest but significant elevations in the Hill coefficient, (n_H_) which is typical of monomeric systems^34^ (Supplementary Fig. S5). It is apparent that since both Spike S1 proteins contain the same distal RBD domain, the occurrence of either the amino acid aspartic acid (D) or glycine (G) at residue 614 has the ability to modify ACE2 affinity for the shared RBD. The data further indicate that the positive cooperativity property may be intrinsic to the ACE2 interaction with the RBD. The values of K_D_ for each variant are included in Table 1.

**Table 1:** Binding constants K_D_ for ACE2 binding to SARS-CoV-2 spike variant proteins.

Spike variant	K_D_ , ng/ml (± SE)	B_max_ , a.u. (± SE)	Rsq
Original [D614] S1	72 ± 3	12.4 ± 0.2	0.998
α/β/γ [D614G] S1	103 ± 17	11.2 ± 1.1	0.951
αβγ [D614G] RBD	84 ± 6	13.0 ± 0.5	0.991
Mink [Y453F] RBD	30 ± 3	16.1 ± 0.6	0.981
α/β/γ [N501Y] RBD	25 ± 1	14.7 ± 0.2	0.997
β [E484K] RBD	94 ± 20	10.3 ± 1.1	0.955

**Figure 2:**
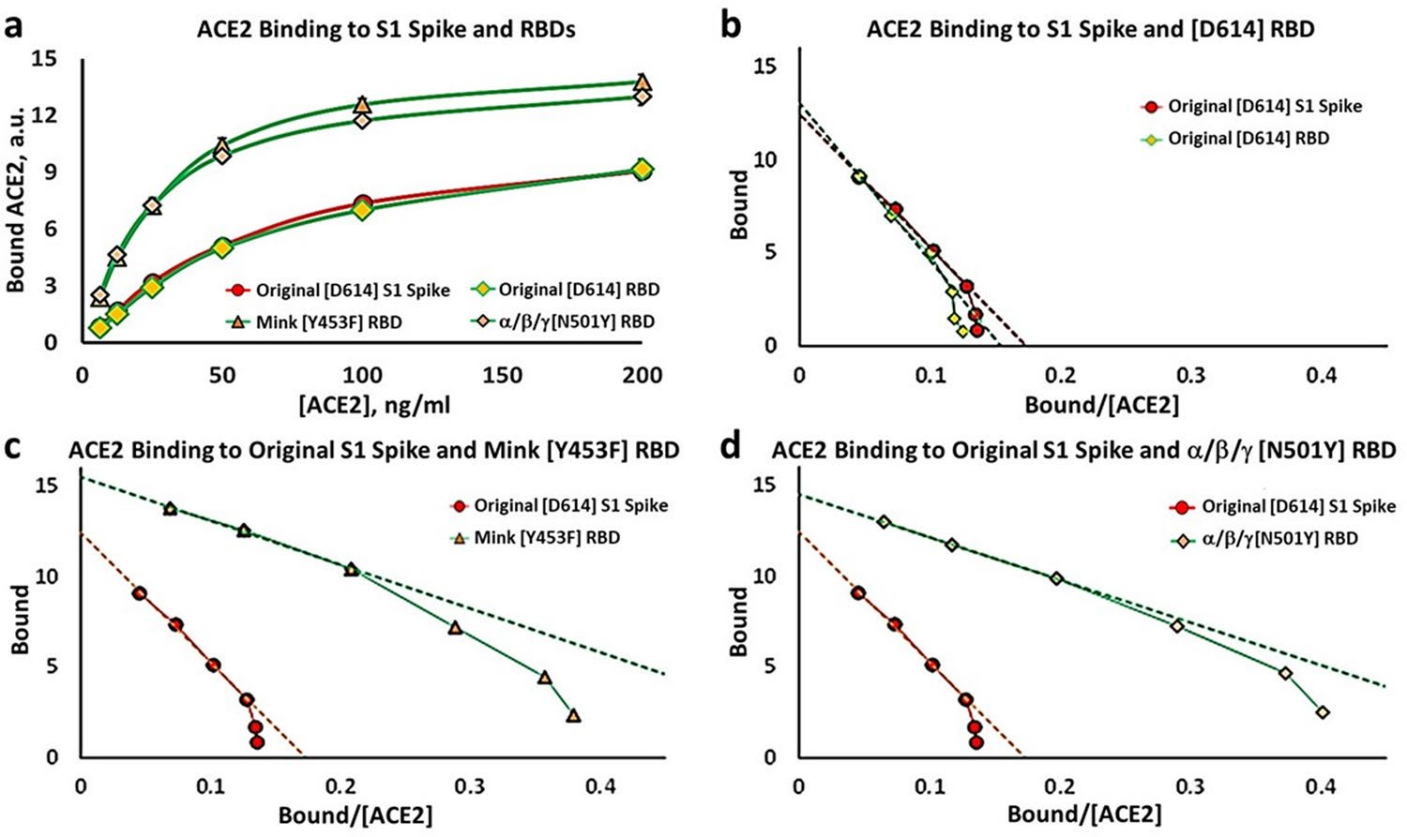
Binding kinetics for ACE2 to original [D614] and mutant spike variants. (**a**) Substrate-Binding plots for ACE2 binding to original [D614] S1, original [D614] RBD, Mink [Y453F] RBD and α/β/γ [N501Y] RBD proteins. (**b**–**d**) Eadie–Hoffstee plots of data in *Part (a)* for original [D614] RBD and S1 spike (**b**), original [D614] S1 spike and Mink [Y453F] RBD (**c**), and original [D614] S1 spike and α/β/γ [N501Y] RBD (**d**). A *reduction* in slope corresponds to a *reduction* in K_D_, and thus an *increase* in affinity. Each point is the average ± SE for N = 5–6 independent experiments.

We also tested ACE2 binding properties to RBD proteins containing three other disease-associated mutations, and compared them to the Original [D614] RBD. Figure 2a shows the substrate-binding isotherms for the Mink [Y453F] RBD and the α/β/γ [N501Y] RBD proteins, and compares them with the Original [D614] S1, and the Original RBD proteins. Figure 2b shows the Eadie–Hoffstee plots for the Original [D614] S1 and the Original RBD. The Original RBD domain is contained within the Original [D614] S1 protein, and as expected the data are virtually superimposable. Both curves are concave down at low ACE2 binding levels, and thus are consistent with the interpretation of positive cooperativity. Figure 2c, d show the same type of data for the Mink [Y453F] RBD protein and the α/β/γ [N501Y] RBD protein, respectively. The Eadie–Hoffstee plots are concave down at low values of binding, while the slopes in the linear parts of the plots are substantially *reduced* compared to the Original S1 proteins. As summarized in Table 1, K_D_ values for the Mink [Y453F] RBD and α/β/γ [N501Y] RBD variants are significantly reduced from 72 to 30 and 25 ng/ml ACE2, respectively. Thus mutations in the RBD in the region interacting with ACE2 can affect the K_D_ for the interaction.

**Figure 3:**
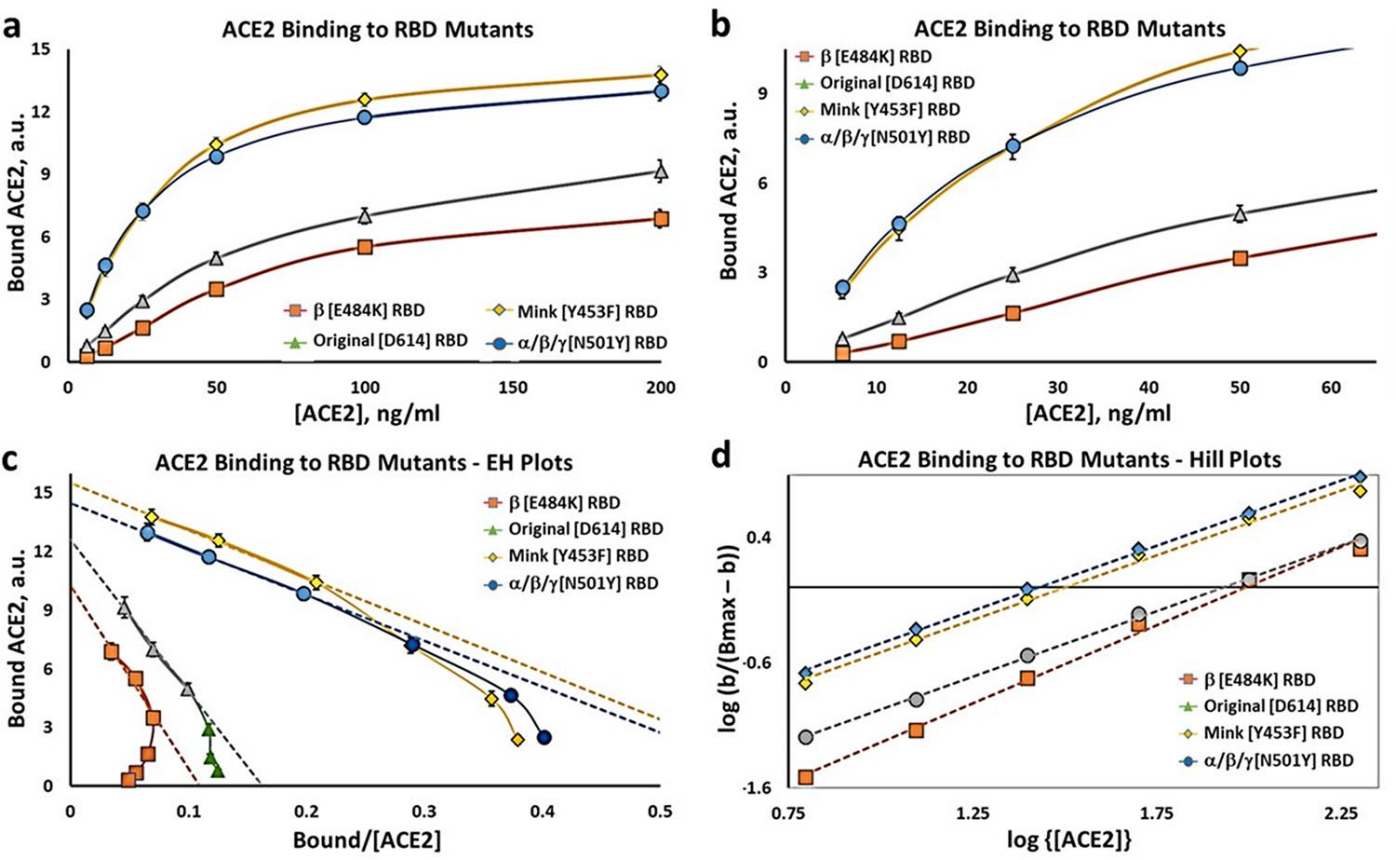
Binding kinetics for ACE2 to β [E484K], Mink [Y453F], α/β/γ [N501Y], and original [D614] RBDs. (**a**) Substrate-binding plots for ACE2 binding to RBD proteins. (**b**) Data from *Part* (*a*) at low concentrations of ACE2. (**c**) Eadie–Hoffstee plots for mutant RBD proteins compared to Original [D614] RBD. (**d**) Hill plots for mutant and Original [D614] RBD proteins. Each point is the average ± SE for N = 5–6 independent experiments.

Figure 3a shows the substrate-binding plots for the recombinant β [E484K] RBD protein. Compared to the other RBD variants the β [E484K] RBD has a slightly more evident lag than the original Wuhan RBD. The ACE2 concentration region of 6.25-to-50 ng/ml is expanded in Fig. 3b to emphasize this comparison with other variant RBD proteins. To further test for the ACE2 positive cooperativity mechanism we analyzed the β [E484K] RBD data using an Eadie–Hoffstee plot (Fig. 3c). As for the other RBD variants the β [E484K] RBD protein has a profound "concave-down" structure for the lower values of ACE2 binding. The B_max_ is similar to that of the other variants, but the binding constant, K_D_, trends higher (Table 1). The Hill plot for the β [E484K] RBD protein is shown in Fig. 3d. The slope for the β [E484K] RBD variant, (n_H_), is 1.25 ± 0.05, and is significantly higher than for the other RBD proteins (Supplementary Fig. S5). The elevated Hill constant is consistent with the relatively exaggerated concavity of the EH plot at low levels of ACE2 binding. Thus both the cooperativity and the affinity of ACE2 for the receptor binding domain can be modified by mutations not only in the RBD domain itself, but also by mutations elsewhere in the S1 region of the spike protein.

Sigmoid substrate-binding plots can also be due to autocatalysis, such as can occur during phase transitions. Therefore, to test for whether ACE2 binding to the Spike S1 derivatives might be due to autocatalysis as opposed to positive cooperativity, per se, we analyzed the ACE2 binding data using the recently reported Dhatt, Banerjee and Battacharrya (DBB) plot^35^ (Supplementary Fig. S2). In this plot pure Michaelis–Menten kinetics would be a straight horizontal line parallel to the X-axis. By contrast, autocatalysis would be a straight angular line, and positive cooperativity would be non-linear. The data show that the relationships are non-linear, thus corresponding to positive cooperativity. Importantly, the data at any point define the apparent K_D_, and serve to show that the K_D_ values at higher concentrations of ACE2 are similar to values deduced from the linear portions of the Eadie–Hoffstee plots.

### Cardiac glycoside drugs are competitive inhibitors of ACE binding to the RBD

**Figure 4:**
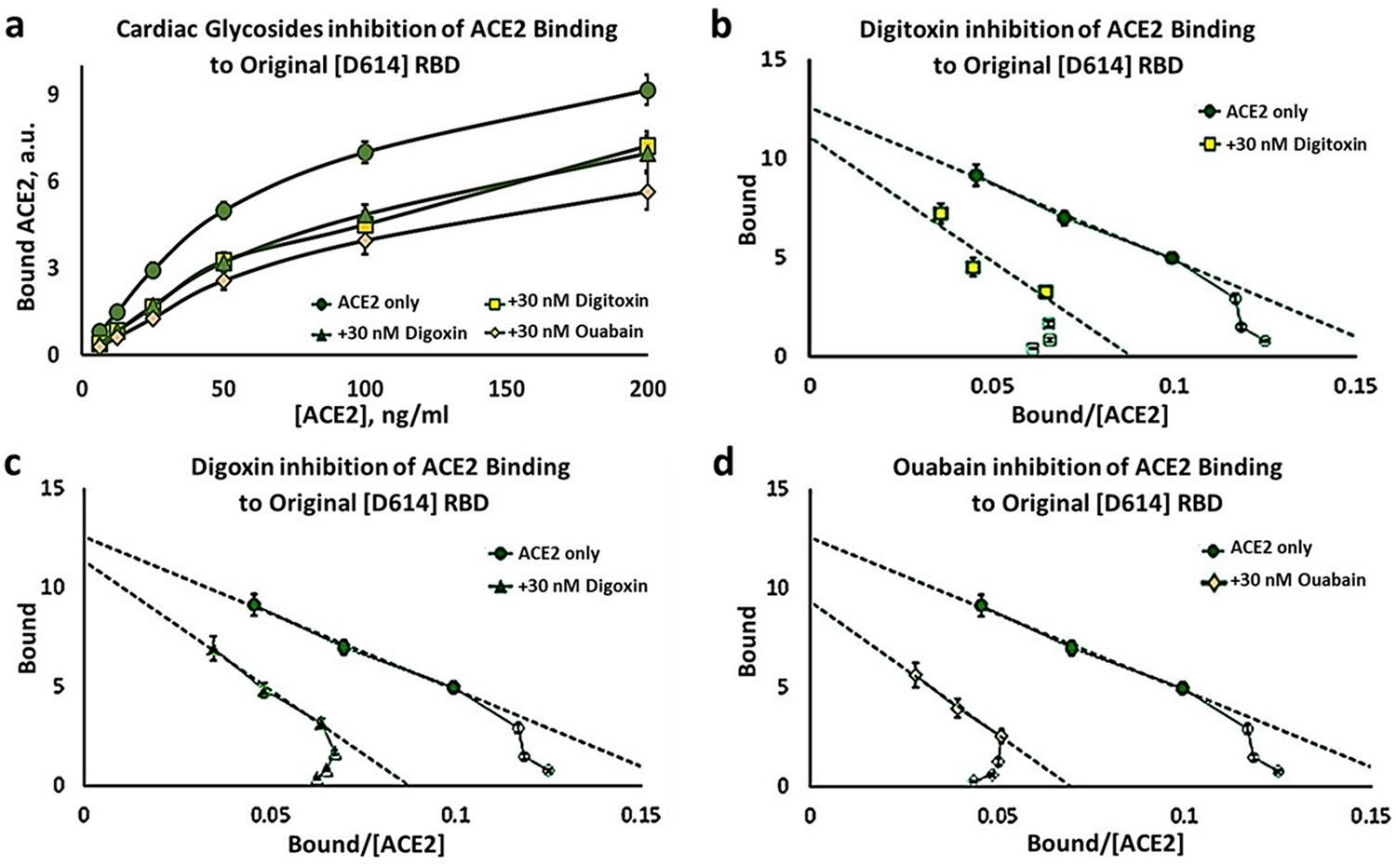
Inhibition of ACE binding to the Original [D614] RBD by cardiac glycoside drugs. (**a**) Substrate-binding plots for inhibition by digitoxin, digoxin and ouabain (30 nM). (**b**–**d**) Eadie–Hoffstee plots of data in *Part* (*a*) for digitoxin inhibition (**b**), digoxin inhibition (**c**), and ouabain inhibition (**d**). Each point is the average ± SE for N = 5–6 independent experiments.

To test for the ability of cardiac glycosides to inhibit ACE2 binding to the RBD protein, we measured ACE2 binding to the Original RBD protein in the presence of digitoxin, digoxin and ouabain (Fig. 4a). Reduced ACE2 binding in the presence of these drugs is evident. The substrate-binding isotherms also indicate that the sigmoid appearance is sustained. Thus the positively cooperative binding mechanism is still present. The DBB plots also provide evidence for a sustained positive cooperativity mechanism for ACE2:RBD binding in the presence of the cardiac glycoside drugs (see Supplementary Fig. S3).

**Figure 5:**
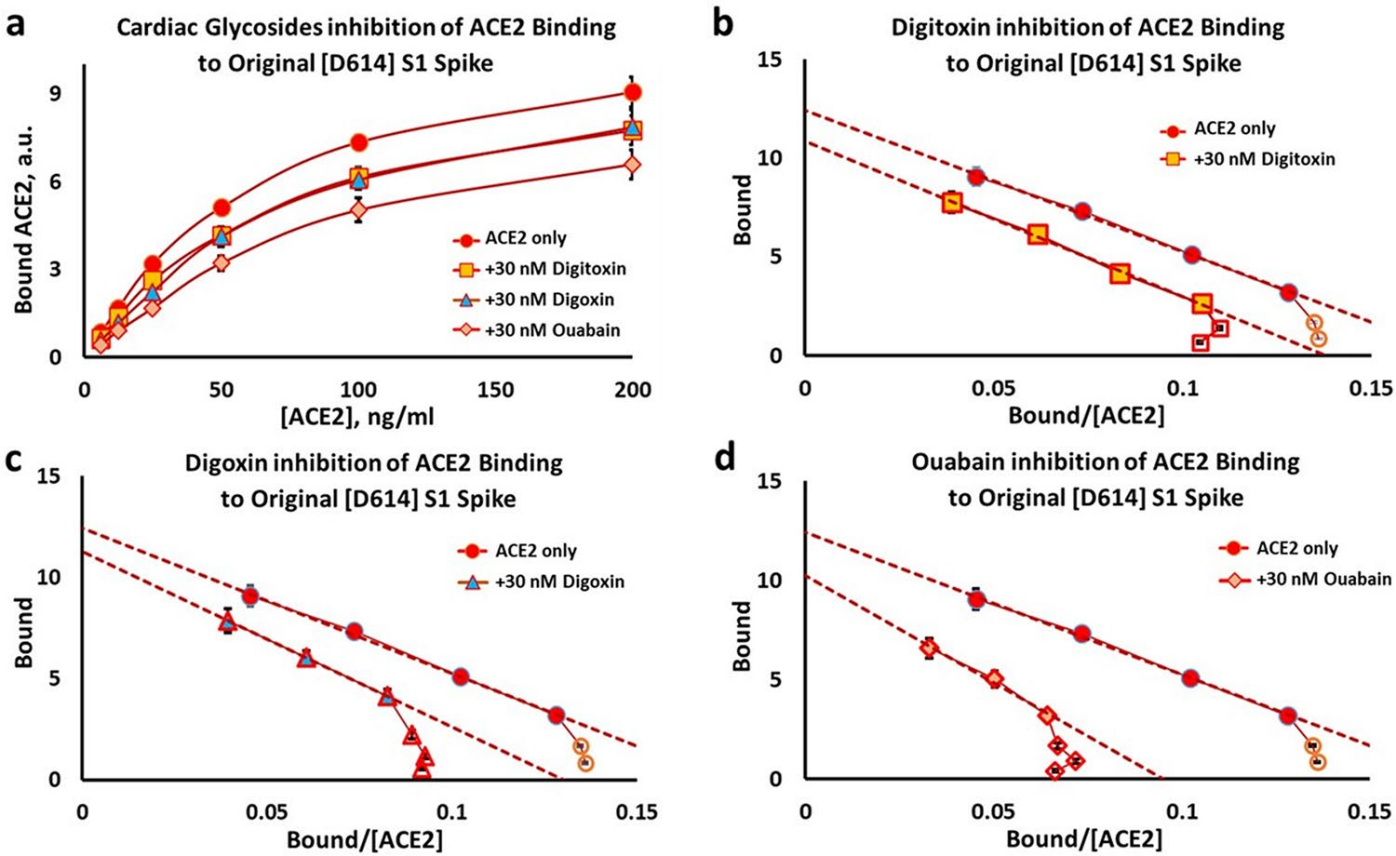
Inhibition of ACE binding to the Original [D614] S1 spike by cardiac glycoside drugs. (**a**) Substrate-Binding plots for inhibition by digitoxin, digoxin and ouabain (30 nM). (**b**–**d**) Eadie–Hoffstee plots of data in *Part* (*a*) for digitoxin inhibition (**b**), digoxin inhibition (**c**), and ouabain inhibition (**d**). Each point is the average ± SE for N = 5–6 independent experiments.

**Figure 6:**
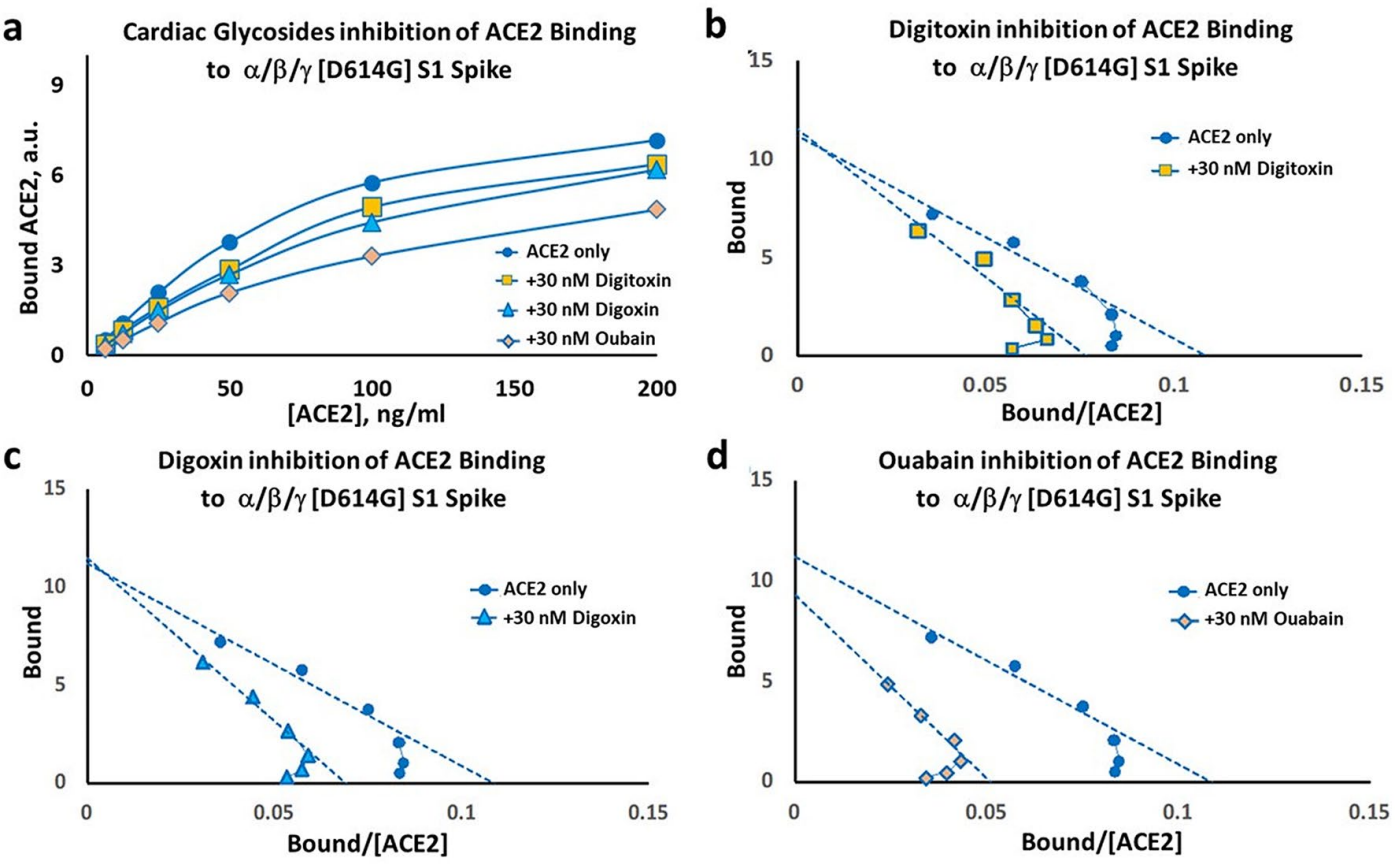
Inhibition of ACE2 binding to the α/β/γ [D614G] S1 spike by cardiac glycoside drugs. (**a**) Substrate-Binding plots for inhibition by digitoxin, digoxin and ouabain (30 nM). (**b**–**d**) Eadie–Hoffstee plots of data in *Part* (*a*) for (**b**) digitoxin inhibition; (**c**) digoxin inhibition; and (**d**) ouabain inhibition. Each point is the average ± SE for N = 5–6 independent experiments.

In addition, the Eadie–Hoffstee plots for all three drugs show that the cardiac glycosides differentially increase the K_D_ values while keeping the B_max_ values approximately constant (see Fig. 4b–d). Similar data were obtained for the Original [D614] S1 protein (Fig. 5) and for the α/β/γ mutant [D614G] S1 protein (Fig. 6). These data are consistent with a competitive inhibition mechanism. The individual EH plots show that these drugs all appear to lower the affinity of ACE2 by 50–100% while keeping the B_max_ approximately constant. These kinetic data are summarized in Table 2, and indicate that of the three drugs tested ouabain is the most potent inhibitor, followed by digitoxin and digoxin.

**Table 2:** Cardiac glycoside inhibition of ACE2 binding to SARS-CoV-2 spike variant proteins.

Spike variant	Inhibitor K_i_, nM (± SE)				
	Digitoxin	Digitoxigenin	Digoxin	Digoxigenin	Ouabain
Original [D614] S1	69 (± 8)	63 (± 6)	53 (± 6)	35 (± 3)	24 (± 2)
α/β/γ [D614G] S1	71 (± 13)	54 (± 5)	48 (± 5)	34 (± 4)	21 (± 2)
α/β/γ/[D614G] RBD	28 (± 3)	27 (± 2)	29 (± 2)	21 (± 2)	16 (± 1)
β [E484K] RBD	43 (± 13)	60 (± 33)	62 (± 23)	30 (± 4)	21 (± 4)
Mink [Y453F] RBD	71 (± 12)	41 (± 4)	67 (± 12)	26 (± 2)	45 (± 5)
α/β/γ [N501Y] RBD	62 (± 11)	39 (± 5)	43 (± 5)	23 (± 2)	37 (± 4)

Values are based on calculating K_i_ values for all concentrations of ACE2 and averaging all values for the individual recombinant proteins. Each point is the average ± SE for 5–6 independent experiments.

Digitoxin and digoxin have three sugars on their 3'-OH groups, while the more potent inhibitor ouabain has only one sugar (see Supplementary Fig. S1b). We therefore tested whether the sugar moieties on digitoxin or digoxin contributed to inhibitory activity. Digitoxigenin and digoxigenin are the sugar-free derivatives of digitoxin and digoxin, respectively. We found that for ACE2 binding to the Original S1 spike protein, 30 nM digitoxigenin was approximately as inhibitory as parental digitoxin (Supplementary Fig. S4a). A similar result was noted for digoxin and digoxigenin (Supplementary Fig. S4b). We also found similar results for inhibition of ACE2 binding to the α,β,γ mutant [D614G] S1 (Supplemental Fig. S4c and S4d), and to the Original RBD protein (Supplementary Fig. S4e and S4f). The DBB plots in Supplementary Fig. S3 also show that the sugar-free forms of digitoxin and digoxin are very similar inhibitors to their respective parental drugs, and that the positive cooperative behavior of the ACE2 binding isotherm is preserved. Additionally, Supplementary Fig. S4 indicates that the blocking mechanism for both sugar-free drugs is by competitive inhibition. The K_i_ values for digitoxigenin and digoxigenin are included in Table 2. The data therefore suggest that the pharmacophore for digitoxin and digoxin inhibition of ACE2 binding to the RBD may reside in their respective steroid nuclei.

**Figure 7:**
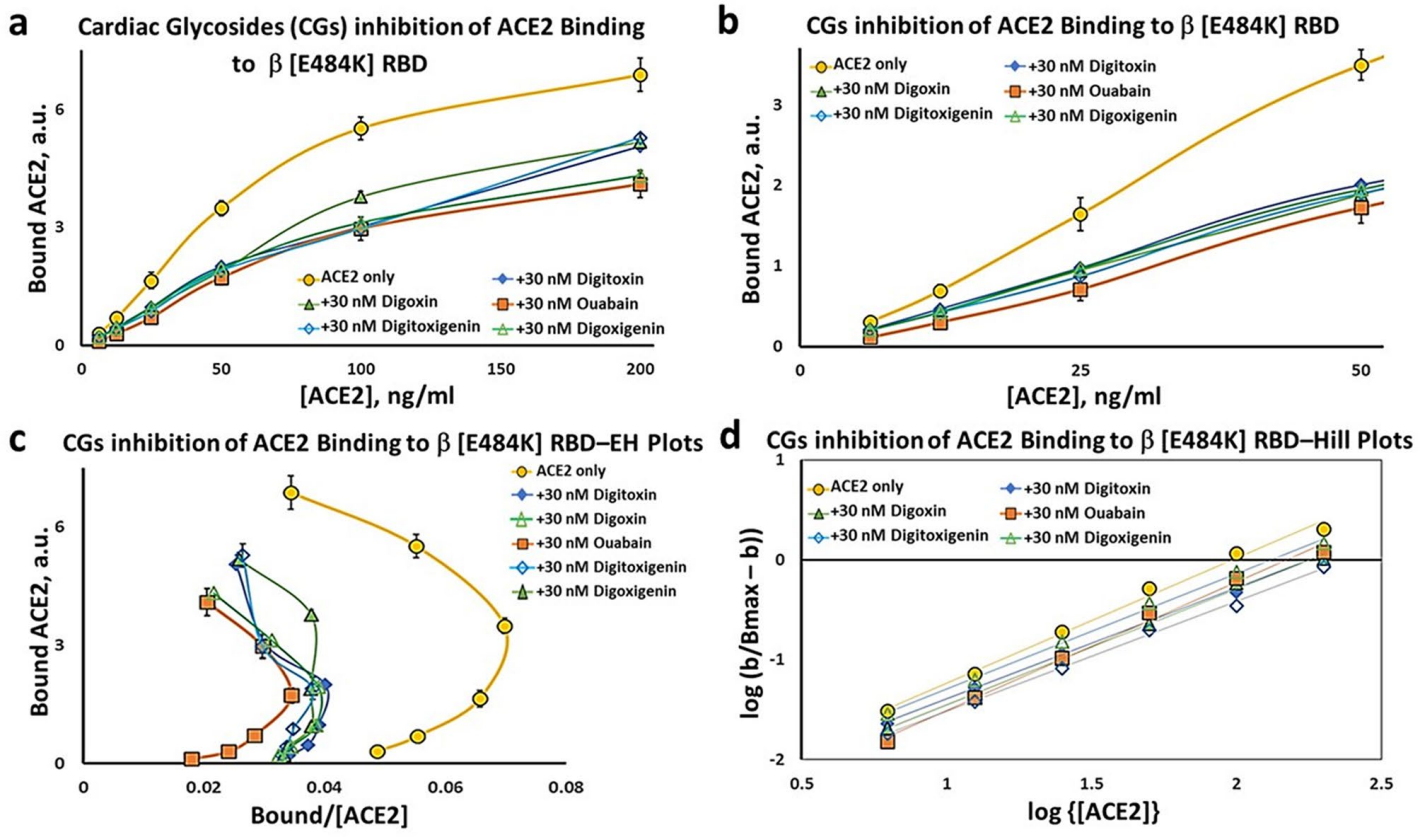
Inhibition of ACE2 binding to β [E484K] RBD by cardiac glycosides. (**a**) Substrate-binding plots for inhibition of ACE2 binding to β [E484K] RBD by digitoxin, digoxin, ouabain, digitoxigenin and digoxigenin (30 nM). (**b**) Substrate-Binding plots of data from *Part* (*a*) at low concentrations of ACE2. (**c**) Eadie–Hoffstee plots of data in *Part* (*a*). (**d**) Hill plots for each titration from *Part* (*a*). Magnitude and error for Hill coefficient (n_H_) given in Supplemental Fig. 5 Each point is the average ± SE for N = 5–6 independent experiments.

Finally, we tested the ability of cardiac glycoside drugs to inhibit ACE2 binding to the β [E484K] RBD protein, the α/β/γ [N501Y] protein and the Mink [Y453F] RBD protein. Figure 7a shows the Substrate-Binding plots for ACE2 binding to the β [E484K] RBD protein. The inhibitory effects are clearly apparent for the cardiac glycoside drugs ouabain, digitoxin and digoxin, and the two sugar-free derivatives, digitoxigenin and digoxigenin. The sigmoid character of the ACE2 binding isotherm is also evident, and is more clearly shown in Fig. 7b for the low ACE2 concentrations. Figure 7c shows the Eadie–Hoffstee plots for both drugs and derivatives. All are concave-down at low concentrations of ACE2. Thus the apparent positive cooperativity for ACE2 binding is preserved in the presence of all three cardiac glycosides and the two sugar-free derivatives. Furthermore, all of these drugs and their sugar-free derivatives have inhibitory activity, although the most active drug is ouabain. Finally, the B_max_ values for these drugs are similar while the slopes of the linear portion of the EH plots at high ACE2 concentration are systematically higher than the control. Thus the inhibitory mechanisms appear classically competitive. Figure 7d shows the Hill plots for all five cardiac glycoside drugs and sugar-free derivatives. Equivalent results for the Mink [Y453F] RBD protein and the α/β/γ [N501Y] RBD protein are shown in Supplementary Figs. S6 and S7 respectively. The calculated K_i_ values for these drugs and their sugar-free derivatives are shown in Table 2. Ouabain has the lowest  K_i_ value of the three cardiac glycoside drugs. Digoxigenin has a slightly lower K_i_ value for Mink [Y453F] and α/β/γ [N501Y]. Thus relative cardiac glycoside potency for inhibition of ACE2:RBD binding appears to depend both on the drug structure, and on the mutation.

The Hill plot slopes, n_H_, from Fig. 7d and Supplemental Figs. S6d and S7d vary between ~ 1.1 and ~ 1.3 for all tested variant Spike proteins, and are summarized in Supplementary Fig. S5a. The highest values are for β [E484K], regardless of which drug or drug-derivative is present in the analysis. To determine the significance of these observed differences we calculated p-values for the difference between the observed n_H_ value for each condition and n_H_ = 1.0, where cooperativity is zero (Supplemental Fig. S5b). The mutant with the highest significance, independent of the treatment, is the β [E484K]. The drug that most significantly affects the most mutants is ouabain. Operationally, a high value of n_H_ is associated with a more lengthy lag in ACE2 binding at low concentrations of ACE2.

### Cardiac glycosides block penetration of Spike-pseudotyped VSV virus into lung cells

**Figure 8:**
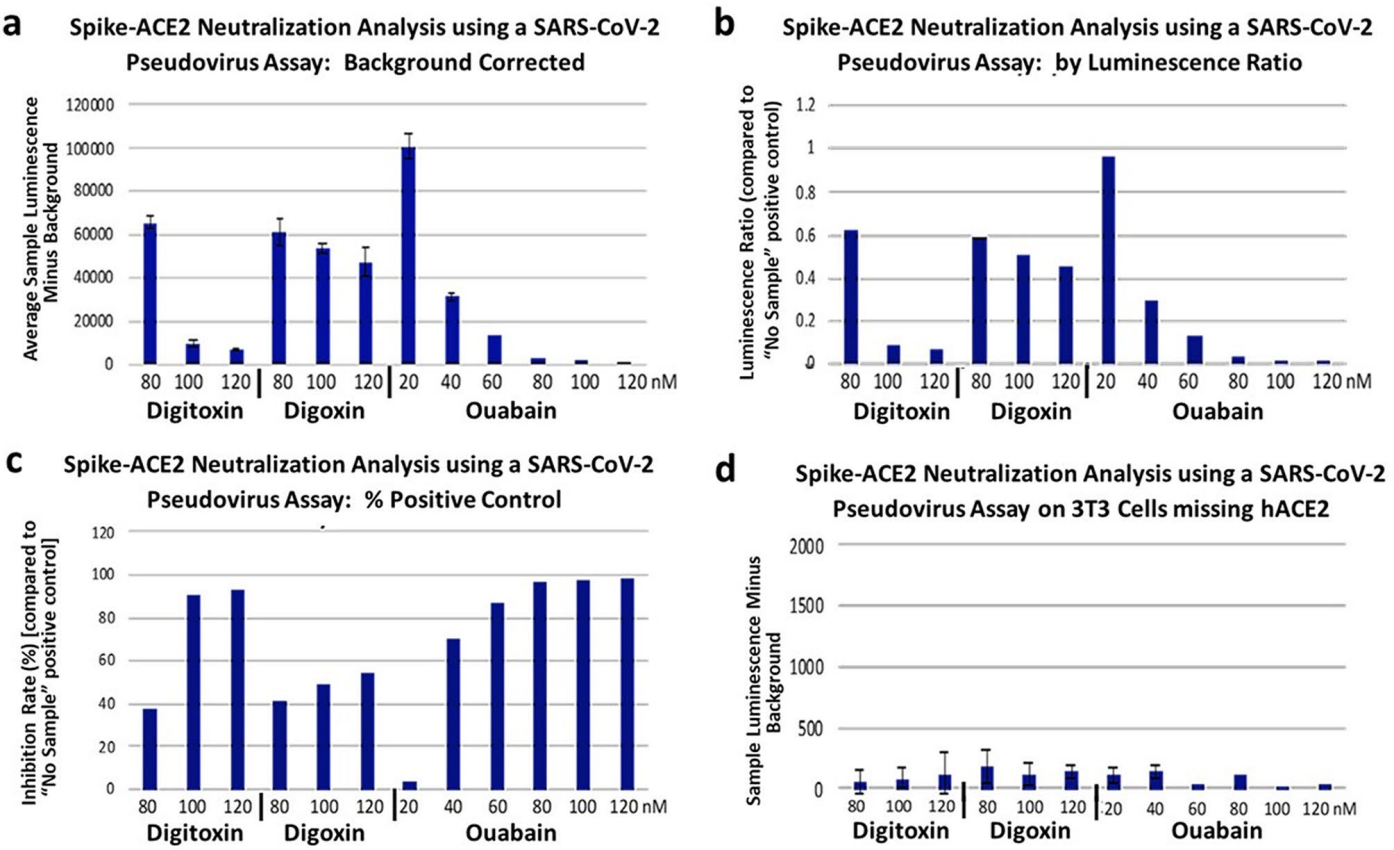
Inhibition of Spike-pseudotyped VSV(S) penetration into human A549 lung cell by cardiac glycosides. (**a**) Measurement of inhibition by luminescence background subtract. VSV(S) was pre-treated with drugs for 1 h, and then exposed to cells in DMEM for 2 h. VSV(S) mixture was then replaced by whole medium and incubated for 24 h. Data are the averages of N = 3 independent experiments. Error bars are ± SE. (**b**) Measurement by luminescence ratio. (**c**) Measurement by % of positive control. (**d**) Negative control with mouse 3T3 cells missing hACE2, measured by luminescence background subtract method.

To test whether cardiac glycosides blocked the viral penetration process we tested drug effects on cell entry by luciferase (*luc*)-loaded Spike-pseudotyped VSV(S) viral particles. To further focus the experiment on the earliest entry steps we also used a triple tandem time-of-addition method. Briefly, VSV(S) particles were *first* preincubated for 1 h with cardiac glycoside drugs in DMEM at 37 °C. *Secondly*, the particles and drugs were then preincubated with the A549 cells in DMEM for 2 h at 37 °C. *Thirdly*, the viral particles and drugs were washed away and replaced by complete DMEM medium. The A549 cells were then incubated for 24 h at 37 °C before assay for luciferase. Figure 8a shows the results of luminescence background-corrected activity of *luc*-loaded pseudotyped VSV(S) over the active ranges for digitoxin, digoxin and ouabain. Ouabain was found to potently suppress viral entry. Digitoxin was less potent and digoxin was the least potent. Based on these data the Hill plot-based EC-50 value for ouabain was calculated to be 16 ± 1 nM (N = 3, ± SE). The positive and negative control values are given in Supplemental Table S1. The inhibition data are similar when measured either by ratio to the luminescence control (Fig. 8b), or by % of positive control (Fig. 8c). As a negative control, and to test for whether human ACE2 receptor was required for cell penetration, mouse 3T3 cells were substituted for human A549 cells. Mouse 3T3 cells were found to be inactive (Fig. 8d). Finally, as a positive human control, post convalescent serum from an unidentified, recovered COVID-19 patient was found to inhibit penetration of the SARS-CoV-2 spike pseudotyped VSV(S) in a dose-dependent manner (Supplementary Fig. S8). Since the SARS-CoV-2 spike pseudotyped VSV(S) only reports on viral penetration, and is not infectious, the data indicate that ouabain most potently blocks viral penetration into human lung cells, likely at a very early stage of the entry process.

### Cardiac glycosides and sugar-free analogues block infectivity by native SARS-CoV-2

To test whether cardiac glycosides and the sugar-free derivatives digitoxigenin and digoxigenin could also block infectivity, we exposed human lung cells to the SARS-CoV-2 NanoLuc reporter virus in the presence of multiple concentrations of all three of the cardiac glycoside drugs and both of the sugar-free derivatives. In a modification of the method used for the pseudotyped virus experiments, cells were simultaneously exposed to virus and compounds for 1 h, and then washed and incubated in complete medium for 24 h before assay. Thus there was no preincubation of the virus with drugs prior to exposure to cells. The data show that ouabain, digitoxin and digoxin inhibit infectivity by 100%, with ouabain and digitoxin being the most potent and digoxin less potent (Supplementary Fig. 9a,b,c,f). The EC50 for ouabain is 36 nM. The sugar-free derivatives digitoxigenin and digoxigenin, are all inhibitors of infectivity, and at maximum concentrations, both inhibit infectivity by 100%. However, these derivatives are significantly less potent than their parent drugs (Supplemental Fig. 9d,e,f). Thus the cardiac glycoside drugs ouabain, digitoxin and digoxin are similarly inhibitory in both the in vitro RBD:ACE2 binding assays, and in the in vivo assays with either Spike pseudotyped virus or native SARS-CoV-2 virus on human A549 cells.

## Discussion

For the SARS-CoV-2 virus to infect a target cell, the Receptor Binding Domain on the viral Spike protein must bind to the receptor protein ACE2 on the cell surface. However, fully understanding the factors controlling this binding process is only at an early stage. Here we have tested the hypothesis that cardiac glycoside drugs such as ouabain, digitoxin, and digoxin might block the binding reaction between ACE2 and the Spike (S) protein, and thus block viral penetration into human lung cells. In this paper both parts of this hypothesis have been experimentally validated in three overlapping tests. Firstly, we found that ACE2 binding to various spike variants occurred by a positively cooperative mechanism. Not unexpectedly, we also found that mutations in the RBD, specifically Mink [Y453F] and α/ β/γ [N501Y], significantly *increased* ACE2 binding affinity, while the β [E484K] mutation trended towards *reduction* in ACE2 binding affinity. We also validated a previous finding that the α/β/γ [D614G] mutation, on a part of the spike S1 protein downstream of the RBD, also reduced ACE2 binding affinity to the RBD. Thus the effect of a mutation that enhances infectivity need not necessarily increase the affinity of ACE2 for the RBD. Secondly, as hypothesized, we found that ouabain, digitoxin and digoxin were high-affinity competitive inhibitors of ACE2:RBD binding. In addition, we found that the *relative inhibitory potencies of these drugs, as defined by the K*_*i*_*, were mutation- and drug-dependent.* Lastly, we found that ouabain and digitoxin, but less so digoxin, both potently blocked SARS-CoV-2 Spike pseudotyped virus penetration into human lung cells, and also blocked infectivity by native SARS-CoV-2. This was in spite of (i) two different lines of engineered A547 cells; (ii) two different kinds of virus; (iii) performance in different laboratories, in different institutions; and (iv) different protocols for drug testing. Inasmuch these drugs are widely available, and are clinically safe for those with normal hearts^33^, it is quite possible that these common cardiac medications could be repurposed for anti-COVID-19 therapy.

In retrospect the positively cooperative mechanism by which soluble monomeric ACE2 binds to the RBD might have been anticipated. For example it has been recently reported that when either the Original RBD protein or the Original [D614] Spike S1:S2 protein binds to ACE2 there is a 3–tenfold increase in the intrinsic ACE2 carboxypeptidase activity^36^. Thus functional changes in ACE2 can be induced by interaction with Spike proteins. Furthermore, structural analysis by Cryo-EM shows that the binding of the Original RBD to ACE2 induces a ~ 12° movement of the ACE2 C-terminal domain toward the N-terminal domain, and also induces an opening of the ACE2 substrate binding pocket^37^. Such changes in conformation are intrinsic to the two major models for positive cooperativity, the "induced fit" model^34,38,39^ and the earlier "sequential" or "symmetry" model^34,40^. Furthermore, the modest Hill coefficients shown here for ACE2:RBD interactions are also consistent with enzymatic and biophysical data previously reported for positively cooperative monomeric systems such as glucokinase^41^ and trypsin-like proteases^42^. These data for ACE2:RBD binding might therefore be consistent with an "induced fit" hypothesis, in which ACE2 could initially bind to the RBD with low affinity, and then undergo further conformational changes to accomplish a higher affinity interaction. On the other hand, dimers of native ACE2 have been shown to be preferred as a binding partner for the RBD for a pseudotyped SARS-CoV-2 spike^37^. Consistently, monomers of ACE2 have been reported to bind poorly to intact viral Spikes, compared to the binding by ACE2 dimers. In addition, it has been reported that more than one RBD in a triple protomer spike can elevate into the open state and bind to multiple host ACE2 extracellular domains^43,44^. It has also been reported that the binding of soluble, nominally monomeric ACE2 to the biologically intact spike protein is also positively cooperative, with the expected modest values for the Hill coefficient^10^. Finally, recent in silico modeling has suggested that higher concentrations of recombinant extracellular ACE2 might trend towards a dimeric polymerization state^45^. We conclude that further analysis will be necessary to distinguish whether monomeric or dimeric ACE2 models, or both, depending on concentration, might best describe the experimental data for isolated domains of the Spike protein.

The parallel results with cardiac glycoside drugs are all the more remarkable when considering the relative simplicity of the in vitro RBD:ACE2 binding assay, with the comparative complexity of the two types of in vivo experiments with spike-pseudotyped and native SARS-CoV-2. It is a consideration that the spike pseudotyped VSV and the SARS-CoV-2 strains both possessed original [D614] sequences, and might best be compared to the original RBD and the original [D614] S1 in Table 2. However, regardless of the mutations tested in vitro, ouabain sustained its high potency when compared to all three of the cardiac glycoside drugs in all three systems. By contrast, the potency of digitoxin and digoxin in the in vitro ACE2:RBD binding assays depended on the mutations present in the RBD, and on the presence of the [D614G] mutation in the S1 domain. However, digitoxin was approximately equally potent to ouabain in the in vivo assay with SARS-CoV-2, and more potent than digoxin in both in vivo assays. The relatively low potency of digoxin compared to ouabain was also reported by Cho et al.^23^ in studies with SARS-CoV-2 infectivity of the green monkey kidney Vero-E6 cells.

However, it is puzzling that in the in vivo SARS-CoV-2 assay with digitoxigenin and digoxigenin, these sugar-free derivatives of digitoxin and digoxin, respectively, retained their inhibitory capacity but were much less potent than their parental compounds. One possibility was that other sites in the in vivo assay might have bound these steroids, thereby lowering their availability. Based on wide screens of FDA-approved drugs and related compounds, in silico studies by Carino et al.^30^ identified two steroid binding sites on the original RBD with specificity for triterpenoid steroids and bile acids, respectively. Shoemark et al.^29^ identified a third possible site for steroid binding in the non-RBD free fatty acid pocket with selectivity for dexamethasone. However, neither of these investigators identified their steroid binding sites as having the ability to dock with cardiac glycoside drugs, or with their sugar-free derivatives. Furthermore, Ahmad et al.^27^ identified an in silico docking site for digitoxin and digoxin on ACE2, but not for digitoxigenin or digoxigenin. Wei et al.^26^ did not identify a docking site for digoxigenin in an RBD site for digitoxin binding. Finally, we cannot exclude as yet unknown sites in the complex in vivo reaction mixture that could have contributed to rendering the sugar-free steroids less available. However, insofar as the three cardiac glycoside drugs are concerned, the parallel is sustained between the in vitro ACE2:RBD binding reaction, the in vivo assay for inhibition of viral entry with the spike-pseudotyped VSV system, and the in vivo assay for inhibition of SARS-CoV-infectivity.

Based on data shown here, we have also shown that other regions in the Spike complex might have conformational effects on the RBD and thus its affinity for ACE2. The α/β/γ, [D614G] mutation in the S1 protein may be an example of how changes at some distance from the RBD sequence can raise the K_D_ for ACE2 binding to the RBD. How this happens, and why there are possible advantages to the virus for infectivity, are insights yet to be discovered. However, it has been reported that the α/β/γ, [D614G] mutation increases spike flexibility, and spike density by 4–fivefold^15,16^. Thus in exchange for a reduction in the affinity for ACE2, other properties that enhance infectivity may have been acquired. Nonetheless, the inhibition constants for the cardiac glycosides were not significantly affected by the [D614G] mutation. This is an attractive property for a candidate COVID-19 drug where the virus is constantly mutating and vaccine escape is an ever-present problem.

The anti-viral properties of cardiac glycosides have been well documented, not only for some RNA viruses such as coronaviruses but for some DNA viruses as well^46^. In the case of RNA viruses, ouabain and digoxin have been reported to block native MERS-CoV entry into green monkey kidney Vero cells^24^. Digitoxin has also been reported to have the same effect on MERS-CoV entry into Vero cells^25^. Recent time-of-addition studies on native SARS-CoV-2 with Vero cells have also been interpreted to indicate that ouabain may block viral entry, with an EC_50_ of 24 nM^23^. However, digoxin, with an EC_50_ value of 43 nM, did not appear to block viral entry, and has been hypothesized to interfere with infection at some intracellular site^23^. Consistently, the present analysis directly shows that ouabain and digitoxin, but less so digoxin, act as blockers of viral entry into human lung cells. In sharp contrast, inhibition of viral entry has not been associated with ouabain and other cardiac glycosides on other RNA and DNA viruses. Rather the effects have been generally on downstream activities associated with virus propagation and infection^46^. Thus the specific anti-viral activity of blocking viral entry into cells by low concentrations of ouabain, and to a lesser extent by digitoxin, appears to be limited to the coronaviruses, and here specifically to SARS-CoV-2. Therefore we suggest that the high affinity and mutation-dependent specificity of the ACE2:RBD binding reaction may point to a biologically important process that might be used to therapeutic advantage for COVID-19.

Cardiac glycosides such as digitoxin have a recent history as potent inhibitors of the TNFα-dependent proinflammatory *host response* to influenza virus infection, also known as cytokine storm^47^. Consistently, digitoxin has also been reported to be a potent and efficacious suppressor of TNFα-dependent proinflammatory cytokine expression in cystic fibrosis cells and patients as well^32,48^. Digitoxin also inhibits tumor growth and NFκB signaling in a pre-clinical rat model of castration resistant prostate cancer^49^. Recently, digitoxin was reported to be among the top ten inhibitors of TNFα-activated NFκB signaling in a screen of 2800 FDA approved drugs^50^. The mechanism for digitoxin inhibition of TNFα signaling to NFκB is due to blocking the interaction between the proinflammatory TNFα/TNFR1 complex and the TNFα Associated Death Domain (TRADD) protein^51^. TRADD is the first intracellular adaptor for the activated TNFα/TNFR1 complex. Mutant CFTR also fails to suppress TRADD expression, thus contributing to the explanation for why cystic fibrosis patients present with chronic TNFα-dependent proinflammatory disease in lung and other organs^52^. An interesting property of the TNFα-dependent host response is that digitoxigenin and digoxigenin, the sugar-free analogues of digitoxin and digoxin, respectively, are much less potent as blockers of TNFα/NFκB signaling^53^.

Finally, safety is an issue to be considered, even when treating a lethal disease like COVID-19. As a class, the cardiac glycosides such as digitoxin, digoxin and ouabain are said to have a narrow therapeutic index when treating a patient with heart failure^33^. A narrow therapeutic index means that medicinal dose and the toxic dose are very close, and care must be taken to avoid or control toxicity. However, according to Goodman and Gilman, the authoritative pharmacology text, "…If subjects with normal hearts ingest large but not lethal quantities of digitalis, either in an attempt at suicide or by accident, premature impulses and rapid arrhythmias are infrequent"^33^. Digitalis is an extract of *Digitalis* plant species containing a mixture of digitoxin and digoxin. In support of this recommendation, *normal* subjects have been studied quantitatively after treatment with purified ouabain, digitoxin and digoxin. No toxicity or adverse events have been reported. In specific examples with ouabain, Mason and Braunwald^54^ injected 0.50–0.60 mg ouabain into 12 normal subjects, aged 18–49, and measured forearm vascular resistance and venous tone. No adverse effects were reported. Selden and Smith^31^ administered 0.25 mg ouabain to normal human volunteers, in order to determine ouabain pharmacokinetics. The peak plasma concentration was found to be 12 ng/ml, or 20 nM, with a half-life of 19–24 h. This peak value was slightly more than the EC_50_ of 16 nM measured here for inhibition of virus penetration into human lung cells. These volunteers were also treated daily for 9 days with 0.25 mg ouabain, reaching a plateau in 4–5 days. No adverse events were reported. More recently, *digitoxin* has been administered by mouth to 16 patients with cystic fibrosis (CF), a proinflammatory genetic lung disease^32,48^. Subjects were administered 0.05 or 0.1 mg/day for 28 days in a double blind, placebo controlled, dose-escalation study. Proinflammatory signaling in airway epithelial cells was significantly suppressed at the higher dose, and again no adverse events were associated with the study drug. The peak serum digitoxin concentration in those CF patients who were treated with 0.1 mg/day reached 10 ng/ml, or 13 nM, by day 28. In addition, over the years, multiple studies on digoxin in cardiovascularly normal subjects have been reported^55–58^. In one instance 0.25 mg *digoxin* was administered to cystic fibrosis patients, daily for one week, with no adverse events associated with the study drug^55^. Finally, in a study of 47,884 men, those taking digoxin for heart failure for > 10 years had 25% lower prostate cancer risk (p < 0.001)^59^. Thus toxicity does not necessarily accompany prolonged treatment with these drugs, even for those with cardiac disease. Lastly, and just in case toxicity does occur in cardiac patients or others, Digibind^R^ is now routinely available in poison control centers. Digibind^R^ is an antibody preparation against the sugar-free steroid nucleus common to cardiac glycosides.

It is a limitation of our study that we have not tested whether changes in spike conformation in response to ACE2 binding might also be occurring. Certainly infectivity enhancing mutations such as Mink [Y453F], α/β/γ [N501Y] and β [E484K] profoundly affect ACE2 binding kinetics. But whether RBD conformation intrinsically changes in response to ACE2 binding must be left as a subject for future study. An additional limitation is that while we discovered that the α/β/γ, [D614G] mutation in the S1 protein, which is at some distance from the RBD, raises the K_D_ for ACE2 binding to the RBD, the present data do not reveal the mechanism. We conclude that we must leave this question for the future as well. How this happens, and why there are apparent advantages to the virus for infectivity, are insights yet to be discovered.

## Conclusion

Ouabain, digitoxin and digoxin competitively inhibit ACE2 binding to the SARS-CoV-2 Receptor Binding Domain (RBD), and block virus penetration and infectivity in human lung cells. Clinical concentrations of cardiac glycosides are relatively safe for subjects with normal hearts, and it is therefore possible that these drugs could be repurposed for COVID-19 therapy.

## Materials and methods

### Chemicals and biologics

The drugs digitoxin, digoxin, ouabain, and the sugar-free derivatives digitoxigenin and digoxigenin were purchased from Sigma-Aldrich. Drugs and derivatives were solubilized as 4 mM stock solutions in either 95% ethanol or 100% DMSO, and aliquots were serially diluted in the same solvent, and then into assay medium. In assays containing ethanol or DMSO, the final solvent concentrations were 0.01% or less. With respect to identifying recombinant Spike protein sequences and variants, we have adopted the World Health Organization (WHO) naming system as follows: (i) The Original SARS-CoV-2 virus expressed aspartic acid at position 614 (viz., [D614], and sequences from this original virus are labeled as Original [D614] S1 or Original [D614] RBD. (ii) The [D614G] mutation is present in alpha, beta and gamma strains, and are labeled as α/β/γ [D614G]. (iii) The [N501Y] mutation is present in alpha, beta and gamma strains, and are labeled as α/β/γ [N501Y]. (iv)The [E484K] mutation is present only in the beta strain and is labeled as β [E484K]. Recombinant proteins produced in human T293 cells were obtained as follows: Recombinant (exodomain) Human Angiotensin Converting Enzyme 2 (ACE2) (Cat # 230-30165; lot 04U06020TW) was purchased from RayBiotech (Peachtree Corners, GA, 30092). Human ACE2 Biotinylated Antibody (Cat # BAF933) and Recombinant (Original) Spike S1 RBD protein (Cat # NBP2-90982) were obtained from R&D Systems (Minneapolis, MN, 55413) and NOVUS Biologicals (Centennial, CO, 90982), respectively. Recombinant SARS-CoV-2 (Original) RBD protein (Cat # 40592-VO8H), (Original) Spike [D614] S1 protein (Cat # 40591-V02H), (Original) [D614G] S1 protein (Cat # 40591-V08H3), Mink [Y453F] RBD protein (Cat # 40592-V08H80), (α/β/γ) [N501Y] RBD (Cat #40592-V08H82) and (β) (E484K) RBD proteins (Cat # 40592-Vo8H84) were purchased from SinoBiologicals U.S. Inc. (Chesterbrook, PA 19087).

### Enzyme Linked Immunosorbent Assay (ELISA) for interaction between Spike proteins and ACE2

Purified recombinant SARS-CoV-2 Spike proteins were individually dissolved in coating buffer (16 mM Na_2_CO_3_, 34 mM NaHCO_3_, pH 9.6) at a concentration of 2 μg/ml Spike protein. This solution, in 100 μL aliquots, was then added to wells of Costar 96 well plates (Corning, Corning, NY; Catalog # 2592) and incubated overnight at 4 °C. The next day, wells were washed 3 times in Phosphate Buffered Saline with 0.05% Tween 20 (PBST) at room temperature. Wells were then blocked with 300 μL Blocking Buffer (0.5% Bovine Serum Albumin (BSA), dissolved in PBST) for 2 h at 37 °C. Wells were then washed 3 times in PBST at room temperature (68°F). When cardiac glycosides were to be added, they were dissolved in Reagent Diluent (0.5% BSA in PBST), added in 100 μL volumes to each well, and incubated at 37 °C overnight. The next day, plates were inverted gently on paper towels for 5 min. A volume of 100μL dilutions of ACE2 were added to each well, supplemented with cardiac glycosides as appropriate, and incubated at 37 °C for 2 h. Wells were then washed 3 times with PBST at room temperature. A working concentration of 1 μg/ml human ACE2 biotinylated antibody (R&D Systems, Minneapolis, MN) was prepared, and 100 μL of this solution was added to each well. The plates were protected from light, and incubated at 37 °C for 1 h. Wells were then washed three times in PBST. HRP-Streptavidin (R&D Systems, Minneapolis, MN) was diluted 1:200, and 100 μL of the solution was added. Plates were then incubated at 37 °C for 1 h, and were washed three times in PBST. A substrate solution was prepared from R & D Systems kit components Solution A and Solution B, mixed 1:1, and 100 μL added to each well. Plates were incubated in total darkness for 15 min. A volume of 50 μL Stop Solution (2 N H_2_SO_4_) was then added to each well. The wells were then read at 450 nm and 570 nm on a FLUOstar-Optima scanner (BMG Labtech, GmbH, Ortenberg, Germany). Individual experiments consisted of 3 or 4 technical replicates for each condition. A non-specific control without spike protein was always included in every assay. Except as noted, each experiment was independently repeated 5–6 times. The final results are given as averages ± Standard Errors of all independent experiments.

### Cells and pseudotyped virus

SARS-CoV-2 Spike pseudotyped Vesicular Stomatitis Virus (VSV(S)) particles were generated and tested using pseudovirus rVSV-ΔG-pCAGGS/Spike-luciferase^60–63^ in RayBiotech laboratories (RayBiotech, Peachtree Corners, GA, 30092). Briefly, VSV(S) particles were preincubated in different concentrations of cardiac glycosides in DMEM for 1 h at 37 °C. Thereafter, 100 μL of the mixture was transferred to a semi-confluent culture of A549 human alveolar basal epithelial cells growing at a density of 20,000 cells/well in a 96-well white cell culture plate with a flat clear bottom. Two hours later, VSV(S) particles were removed and the cells were washed 3 times in DMEM. The medium was then replaced with DMEM with complete growth medium containing 10% FBS and 2% penicillin–streptomycin, and the cells were incubated for 24 h at 37 °C/5% CO_2_. To measure luciferase, 50 μL of luciferase substrate replaced the medium in each well. The plate was shaken for 3 min at 300 rpm to lyse cells and equilibrate samples, and luminescence was then measured.

### Cells and SARS-CoV-2

A reporter virus inserted with the nanoluciferase in the SARS-CoV-2 USA-WA1/2020 backbone was obtained from Dr. Pei-Yong Shi’s laboratory (University of Texas Medical Branch, Galveston, TX)^64^ at passage 2 and was further expanded once on Vero E6 at FDA. The virus was tittered using a published method^65^. A549-hACE2 cells were infected with SARS-CoV-2 NanoLuc reporter virus (MOI ~ 0.05) in the presence of compounds for 1 h. After removal of virus and compounds, cells were further incubated for 24 h prior to luciferase assay (mean of n = 3; error bars, S.D.). Cytotoxicity of compounds was determined by measuring the ATP content of treated cells using the CellTiter-Glo Cell Viability Luminescent Assay kit according to the manufacturer’s (Promega) instruction (mean of n = 3; error bars, S.D.).

### Statistics

To determine the significance of changes in kinetic parameters of ACE2 binding to SARS-CoV-2 Spike or RBD mutants we have applied least-squares regression to the linearized Eadie–Hoffstee plot of the binding data, and determined the statistical significance of the difference between the slopes (i.e., K_D_’s) and between the intercepts (i.e., B_max_) using R or Stata statistical packages. K_i_ values were calculated for each data point in the linear range depending on the inhibition mechanism. For pseudovirus experiments, the EC_50_ values for viral entry into cells was calculated by fitting the luminescence data to a modified Hill plot. K_i_ values were calculated from the Hill equation. Except as noted, all plotted data points are the means of 5 or 6 independent experiments. Means ± SE were calculated.

## Supplementary Information

Supplementary Information.
