# Supplementary material for: Common cardiac medications potently inhibit ACE2 binding to the SARS-CoV-2 Spike, and block virus penetration and infectivity in human lung cells
Source: Sci Rep. 2021 Nov 12;11:22195. doi: 10.1038/s41598-021-01690-9 (PMC8589851; doi:10.1038/s41598-021-01690-9)

**SUPPLEMENTARY DATA**

**Common cardiac medications potently inhibit ACE2 binding to the SARS-CoV-2 Spike, and block virus penetration and infectivity in human lung cells**

Hung Caohuy 1,2,*, Ofer Eidelman 1,2,*, Tinghua Chen 1,2,3,, Shufeng Liu 4 , Qingfeng Yang 1, 5, Alakesh Bera 1,2, Nathan Walton 1,2,3,, Tony T. Wang 4 , Harvey B. Pollard 1,2,3,

*= co-first authors: these investigators contributed equally to this work.

1. Department of Anatomy, Physiology and Genetics, Uniformed Services University School of Medicine, Uniformed Services University of the Health Sciences, Bethesda, MD 20814.
2. Collaborative Health Initiative Research Program (CHIRP), Uniformed Services University of the Health Sciences, Bethesda, MD 20814
3. Consortium for Health and Military Performance (CHAMP), Uniformed Services University of the Health Sciences, Bethesda, MD 20814

Uniformed Services University of the Health Sciences, Bethesda, MD 20814

1. Laboratory of Vector-Borne Viral Diseases, Division of Viral Products, Center for Biologics Evaluation (CBER), U.S. Food and Drug Administration, Silver Spring, MD 20993
2. Center for the Study of Traumatic Stress (CSTS), Uniformed Services University of the Health Sciences, Bethesda, MD 20814

*Communications:

Harvey B. Pollard, M.D., Ph.D.

Department of Anatomy, Physiology and Genetics Uniformed Services University School of Medicine,

Uniformed Services University of the Health Sciences, Bethesda, MD 20814 T: 301-295-3200; Email: [harvey.pollard@usuhs.edu](mailto:harvey.pollard@usuhs.edu)

Keywords: COVID-19, SARS-CoV-2, Spike, cardiac glycosides, digitoxin, digoxin, ouabain, induced fit hypothesis, positive cooperativity, pseudotyped


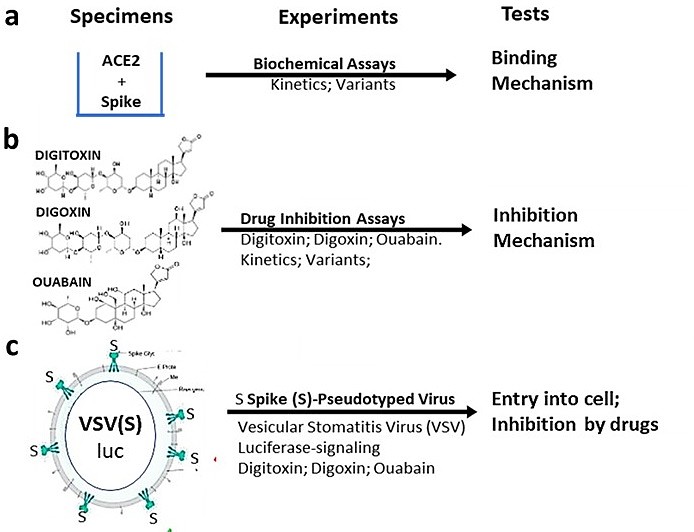


**Supplementary Figure S1**. **Experimental Design. (a)** *In vitro* Biochemical assays. **(b)** *In vitro* drug inhibition assays. **(c)** *In vivo* virus assays with cardiac glycoside drugs/derivatives.


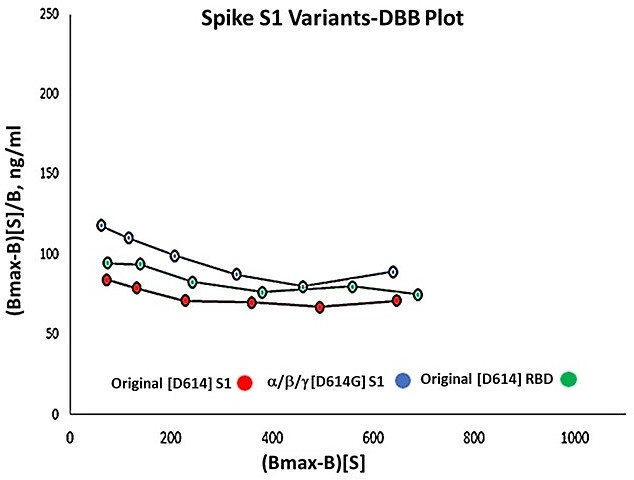


**Supplementary Figure S2. Distinguishing positive cooperativity from autocatalysis using the Dhatt, Banerjee and Battacharrya (DBB) plot for ACE2 binding data.** Validated autocatalytic mechanisms, such as phase transitions, would be straight diagonal lines in the plot. Michaelis-Menten kinetics would be horizontal lines. Cooperative mechanisms would be non- linear. Color codes: Original [D614] S1 (red); α/β/γ [D614G] S1 (blue); Original [D614] RBD (green). Each point is the average ± SE for N=5-6 independent experiments.


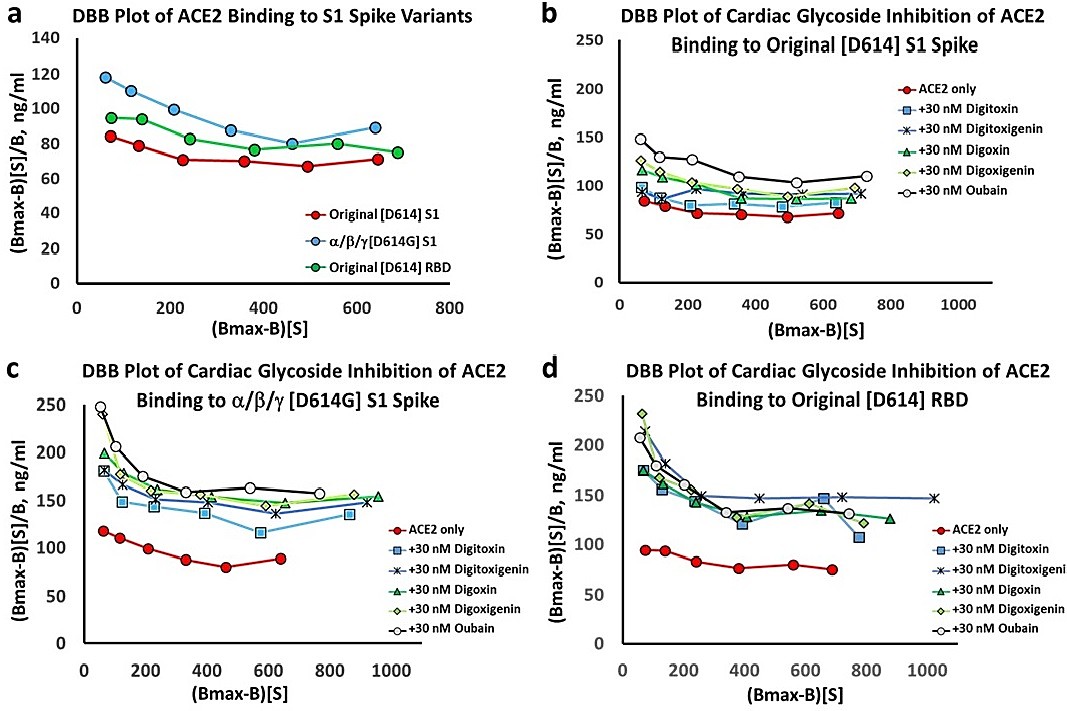


**Supplementary Figure S3**. **DBB plots for inhibition of ACE2 binding to spike proteins by sugar- free cardiac glycosides. (a)** ACE2 binding to Spike variants. (**b**) ACE2 binding to Original [D614] S1 inhibited by 30nM digitoxigenin and digoxigenin, and cardiac glycosides. (**c**) & (**d**): Plots for α/β/γ [D614G] S1 and Original [D614] RBD, respectively. Red color = ACE2 alone. Consistently, elevation on the graph indicates lower KD. Each point is the average ± SE for N = 5-6 independent experiments.


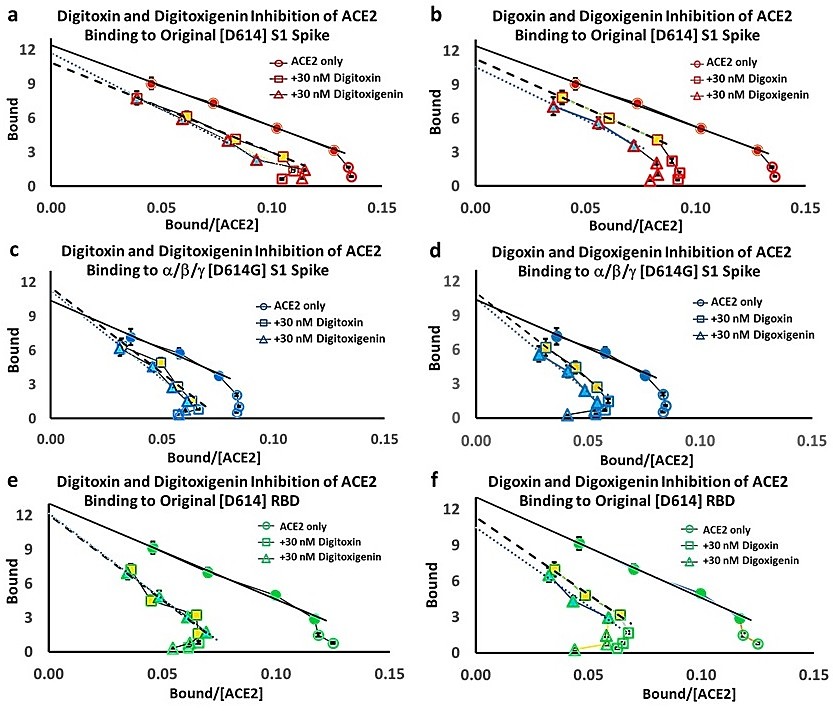


**Supplementary Figure S4**. **Inhibition of ACE2 binding to spike variants by digitoxigenin and digoxigenin**. (**a)** & (**b**) Inhibition of ACE2 binding to Original [D614] S1 spike. (**c)** & (**d**) Inhibition of ACE2 binding to α/β/γ [D614G] S1 spike. (**e)** & (**f**) Inhibition of ACE2 binding to Original [D614] RBD. Each point is the average ± SE for N = 5-6 independent experiments.


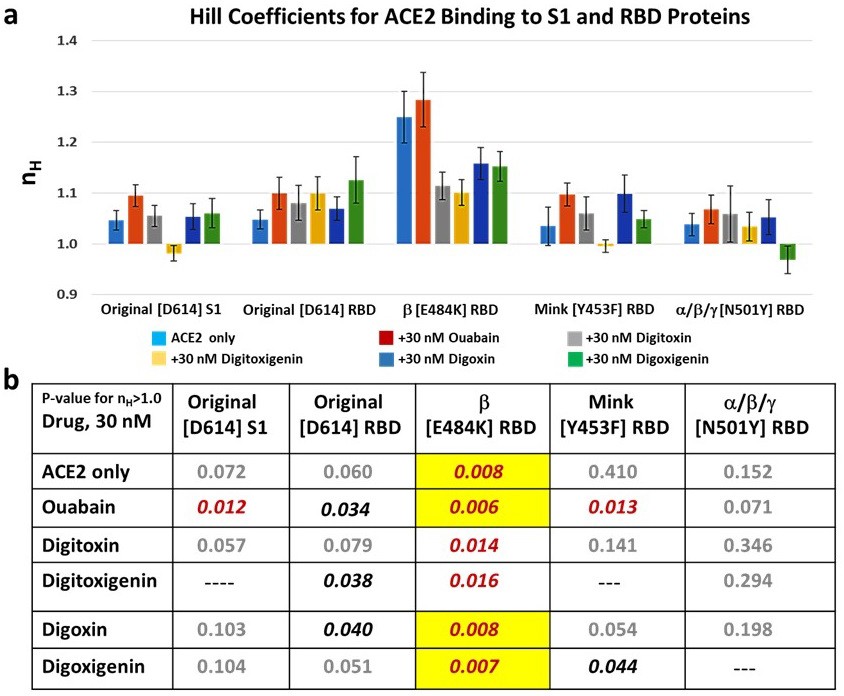


**Supplementary Figure S5. Hill coefficients for cardiac glycoside inhibition of mutant RBD proteins. (a)** Values of the slope, nH, from Hill plots have been calculated for each of the conditions. A value of 1.0 for nH means that there is no cooperativity. A value below 1.0 indicates negative cooperativity. **(b)** *p*-value for the difference of each condition from an nH value of 1.0. Color code: **bold < 0.05; bold red < 0.05**; **highlight <0.01**. The (-) symbol means nH < 1; however, all 3 so marked are not significant.


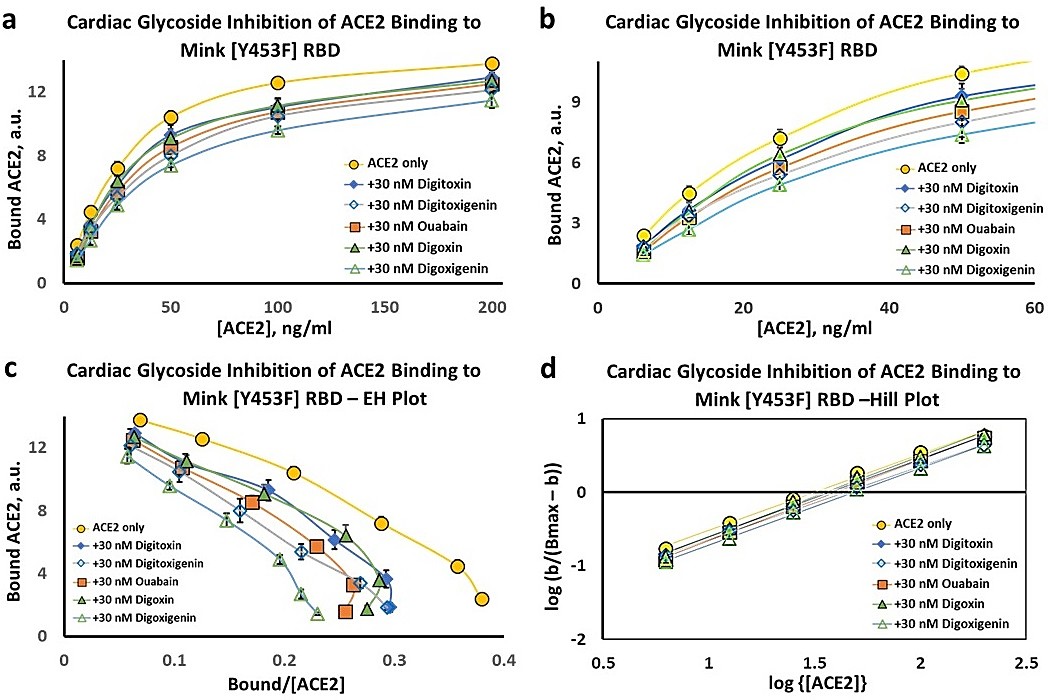


**Supplementary Figure S6. Inhibition of ACE2 binding to Mink [Y453F] RBD protein by cardiac glycoside drugs. (a)** Substrate-Binding plots for inhibition of ACE2 binding to Mink [Y453F] RBD protein by digitoxin, digoxin, and ouabain, (30nM). (**b**) Eadie-Hoffstee (EH) plots for digitoxin inhibition. (**c**) EH plots for digoxin inhibition. (**d**) EH plots for ouabain inhibition. Each point is the average ± SE for N = 5-6 independent experiments.


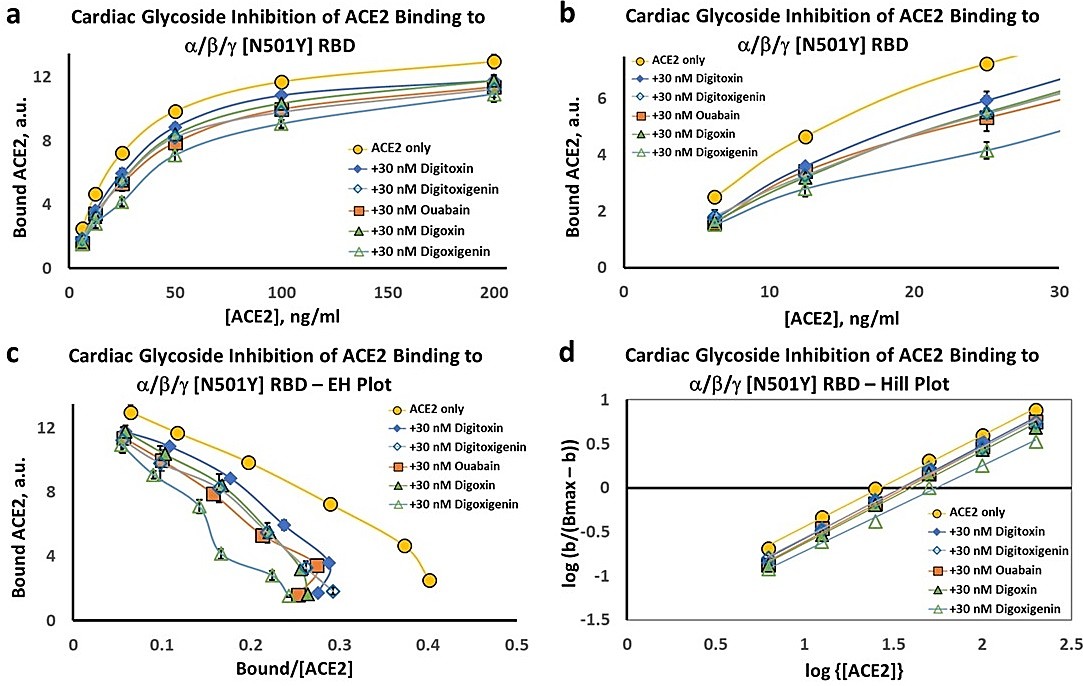


**Supplementary Figure S7**. **Inhibition of ACE2 binding to UK [N501Y]] RBD protein by cardiac glycoside drugs. (a)** Substrate-Binding plots for inhibition of ACE2 binding to α/β/γ [N501Y] RBD protein by digitoxin, digoxin, ouabain, digitoxigenin and digoxigenin (30nM). **(b)** Data from Part (**a**) at low concentrations of ACE2. **(c)** Eadie-Hoffstee (EH) plots of data in Part (**a**). Hill plots for each titration from Part (**a**). Magnitude and error for Hill coefficient (nH) given in **Supplemental Figure 5**. Each point is the average + SE for N = 5-6 independent experiments.


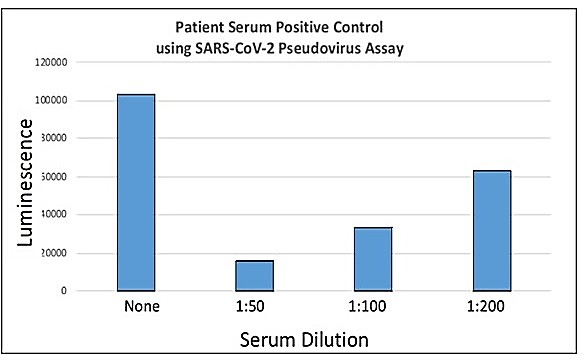


**Supplementary Figure S8. Patient-positive-convalescent serum control using SARS- COV-2 pseudovirus assay.** Recovered COVID-19 patient serum sample was diluted in DMEM and treated as described in methods by the triple tandem time-of-addition method. No cardiac glycosides were added. Control data are summarized in **Supplemental Table 1**.


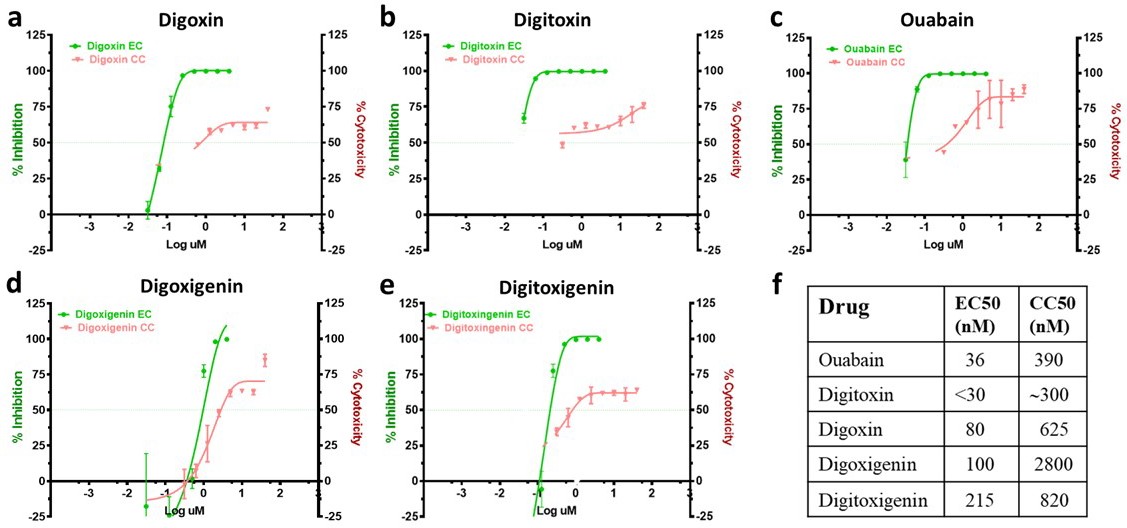


**Supplementary Figure S9. Inhibition of Native SARS-CoV-2 infectivity by cardiac glycoside analogues in hA549 cells. (a), (b) & (c)** Ouabain and digitoxin have very similar potencies. Digoxin is less potent than either digitoxin or Ouabain. **(a) & (d)** Digoxin is more potent than digitoxigenin. **(b) & (e)** Digitoxin is more potent than digitoxigenin. **(d) & (e)** Digitoxigenin is more potent than digoxigenin. **(f)** EC50 and CC50 values for cardiac glycosides and analogues. Cytotoxicity analysis based on ATP content of cells. Symbols: “<” less than; “~” approximately; “EC50” effective concentration for 50% inhibition; “CC50” cytotoxic concentration for 50% cytotoxicity.


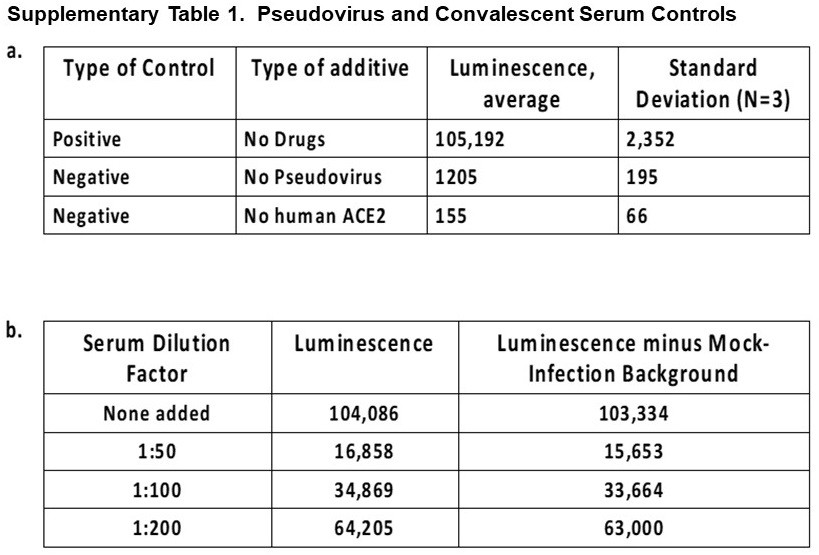

Supplement: Supplementary file 1 — Supplementary Information. [file 41598_2021_1690_MOESM1_ESM.docx]
